# α-Synuclein Interaction with Lipid Bilayer Discs

**DOI:** 10.1021/acs.langmuir.2c01368

**Published:** 2022-08-11

**Authors:** Marija Dubackic, Yun Liu, Elizabeth G. Kelley, Crispin Hetherington, Michael Haertlein, Juliette M. Devos, Sara Linse, Emma Sparr, Ulf Olsson

**Affiliations:** † Physical Chemistry, Department of Chemistry, Lund University, SE-22100 Lund, Sweden; ‡ Center for Neutron Research, National Institute of Standards and Technology, Gaithersburg, Maryland 20878, United States; § Chemical and Biomolecular Engineering Department, University of Delaware, Newark, Delaware 19716, United States; ∥ National Center for High Resolution Electron Microscopy, Centre for Analysis and Synthesis, Chemistry Centre, Lund University, SE-22100 Lund, Sweden; ⊥ Life Sciences Group, Institut Laue-Langevin, 38000 Grenoble, France; # Biochemistry and Structural Biology, Department of Chemistry, Lund University, SE-22100 Lund, Sweden

## Abstract

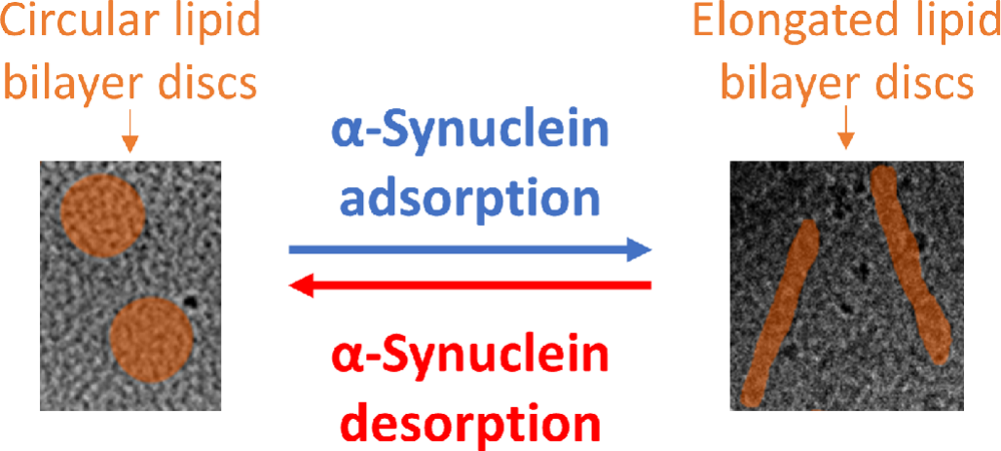

α-Synuclein (aSyn) is a 140 residue long protein present in presynaptic termini of nerve cells. The protein is associated with Parkinson’s disease, in which case it has been found to self-assemble into long amyloid fibrils forming intracellular inclusions that are also rich in lipids. Furthermore, its synaptic function is proposed to involve interaction with lipid membranes, and hence, it is of interest to understand aSyn–lipid membrane interactions in detail. In this paper we report on the interaction of aSyn with model membranes in the form of lipid bilayer discs. Using a combination of cryogenic transmission electron microscopy and small-angle neutron scattering, we show that circular discs undergo a significant shape transition after the adsorption of aSyn. When aSyn self-assembles into fibrils, aSyn molecules desorb from the bilayer discs, allowing them to recover to their original shape. Interestingly, the desorption process has an all-or-none character, resulting in a binary coexistence of circular bilayer discs with no adsorbed aSyn and deformed bilayer discs having a maximum amount of adsorbed protein. The observed coexistence is consistent with the recent finding of cooperative aSyn adsorption to anionic lipid bilayers.

## Introduction

α-Synuclein (aSyn) is a 140 residue natively disordered protein found in presynaptic terminals of neurons. The exact function of aSyn *in vivo* remains mostly unknown. It has been suggested that aSyn plays a role in lipid metabolism,^1−3^ influences phospholipid composition,^4^ and organizes membrane components.^5^ It has further been suggested that aSyn plays a role in neurotransmitter release by promoting membrane remodeling during synaptic transmission.^6^ All of these suggested roles would imply that aSyn–lipid interactions are important for the biological function of the protein.

Under certain conditions, aSyn self-assembles into β-sheet-rich amyloid fibrils, which represent a hallmark of Parkinson’s disease, the second most common neurodegenerative disease.^7−10^ Such fibrils are a main component of intracellular inclusions called Lewy bodies (LBs),^7,8^ which also contain significant amounts of lipids.^11,12^ The observed colocalization of aSyn fibrils and lipids in LBs has stimulated numerous studies of aSyn–lipid interactions.^1,13−24^

It has been shown that the monomeric protein adsorbs to membranes that contain anionic lipids, where it undergoes a secondary structural transformation to an α-helical conformation.^13−16,25^ The ability to adsorb onto lipid membranes may play an important role in the biological function of aSyn.^26^ It was recently inferred that the adsorption of aSyn onto phospholipid membranes occurs with strong positive cooperativity.^17^ Furthermore, it has been found that aSyn may induce vesicle disruption and leakage,^18,19^ as well as vesicle remodelling,^1,20^ i.e., conversion of initially spherical vesicles into micelles of altered morphologies such as cylinders or into bilayer tubes or deformed prolate-shaped vesicles.^17^ The presence of anionic lipid vesicles has been found to accelerate aSyn fibril formation,^21−24^ where it is believed that vesicles with adsorbed aSyn act as a surface for nucleation of fibril formation.^23^

In order to gain more insight into the interactions of aSyn and lipid membranes, we have investigated in the present work a mixture of aSyn and lipid bilayer discs. The discs are composed of a combination of zwitterionic phosphatidylcholine and anionic phosphatidylserine lipids, both with dimyristoyl chains. Here, aSyn adsorption onto lipid bilayer discs was investigated through an adsorption-induced shape deformation of lipid bilayer discs, monitored by cryogenic transmission electron microscopy (cryo-TEM) and contrast-matching small-angle neutron scattering (SANS). The latter experiments were performed with deuterated aSyn and heavy water (D_2_O) buffer, so that the protein was effectively contrast-matched to the solvent, and only the scattering from lipid bilayer discs was visible in the SANS pattern.

## Experimental Section

### α-Synuclein

Protonated aSyn was expressed in-house in BL21 DE3 PLysS Star *Escherichia coli* from a synthetic gene with *E. coli*-optimized codons (purchased from Genscript, Piscataway, New Jersey). An *E. coli* cell pellet containing deuterated aSyn was prepared in the Deuteration Laboratory of the Institut Laue-Langevin (ILL) in Grenoble, France, as previously described.^14^ A high-cell-density fed-batch culture using 85% deuterated Enfors minimal medium was grown with computer-controlled temperature at 30 °C and *p*_O_2__ at 30% saturation.^27^ The protonated and deuterated proteins were separately purified using heat treatment, ion exchange, and gel filtration chromatography, as described previously.^28^ The degree of deuteration was 75%, as determined using mass spectrometry of the purified deuterated protein.

aSyn monomers were isolated using size exclusion chromatography (SEC) in 10 mM MES [2-(*N*-morpholino)ethanesulfonate] buffer (where M = mol/L denotes molar concentration) at pH 5.5 using a 24 mL Superdex75 column (GE Healthcare). The protein concentration was measured using the integrated absorbance at 280 nm of the collected fraction based on the SEC chromatogram and the molar extinction coefficient 5800 M^–1^ cm^–1^.

### Lipid Bilayer Disc Preparation

The lipid bilayer discs were composed of 1,2-dimyristoyl-*sn*-glycero-3-phosphocholine (DMPC) and 1,2-dimyristoyl-*sn*-glycero-3-phospho-l-serine (DMPS), with a molar ratio PC/PS = 7/3. Lipid powders were purchased from Avanti Polar lipids. After weighing each lipid component, the powders were dissolved in a chloroform/methanol mixture (3/1 volume ratio). The solvent was evaporated underneath a stream of N_2_ gas, and the formed lipid film was left in a vacuum oven overnight to be sure that any possible trace of solvent evaporated. The dry lipid film was dispersed in 10 mM MES buffer at pH 5.5. Prior to extrusion, 5 freeze–thaw cycles were performed, where freezing was performed at −20 °C and thawing at 50 °C in the MES buffer at pH 5.5. The thawing was carried out above the expected phase transition temperature for the DMPC/DMPS mixture, which occurs at around 25 °C.^29,30^ The extrusion was performed 21 times through polycarbonate membranes with 50 nm pore size filters using an Avanti miniextruder (Avanti Polar lipids).

### Seeds

We used preformed protonated fibrils at 1 mol % monomer equivalent concentration in order to accelerate protein aggregation. The seeds were formed prior to the experiment by incubating 0.28 mM aSyn monomers in 10 mM MES buffer pH 5.5 under constant stirring. In order to get a dispersion of small seeds, the seed solution was sonicated for 1 min in a bath sonicator prior to mixing with monomers.

### Sample Preparation for Protein–Lipid Mixtures

Salts from the buffer used during SEC were separated from the protein using a 5 mL HiTrap desalting column (GE Healthcare). The collected fractions were lyophilized in order to obtain the high protein concentration required for the scattering experiment. To ensure that the aSyn was initially in its monomeric form, we resuspended lyophilized protein powder in 1 mM NaOH at pH 11.4 (Figure S1). After ca. 30 min, the same volume of 20 mM MES buffer at pH 5.3 was added. Mixing the same volumes of 1 mM NaOH (pH 11.4) and 20 mM MES buffer (pH 5.3) results in 10 mM MES at pH 5.5. Finally, we added a dispersion containing lipid bilayer discs and the sonicated seed solution in 10 mM MES buffer. The final protein concentration was 0.40 mM, and the lipid concentration was 2.1 mM. The samples were incubated at 37 °C, which is above the expected phase transition temperature of the lipids.^29^

### Small-Angle Neutron Scattering

The experiment was performed with the vSANS instrument at NIST Center for Neutron Research (NCNR).^31^ The velocity selector was used to select 6 Å neutrons with a 12% wavelength spread. Two detector banks were used at distances 5 m and 20 m to cover the *q* range from 0.0015 Å^–1^ and 0.13 Å^–1^, where  is the magnitude of the scattering vector, θ being the scattering angle and λ the neutron wavelength. The samples in quartz cells with 1 mm thickness, were mounted on a stage with four sample slots. During the experiment, the samples were slowly rotated to avoid the precipitation of aggregates. The sample stage temperature was controlled to 37 °C and was monitored by measuring the temperature of water in a quartz cell that was mounted to the sample stage. The data collected were reduced to absolute intensity by correcting for the scattering from the background and empty cell following the standard data reduction procedures at the NCNR using the provided IGOR software packages.^32^

### Cryogenic Transmission Electron Microscopy

The morphology of the lipid bilayer discs was examined using a JEM-2200FS transmission electron microscope (JEOL) specially optimized for cryo-TEM at the National Center for High Resolution Electron Microscopy (nCHREM) at Lund University. It is equipped with a Schottky field-emission electron source and operated at an acceleration voltage of 200 kV. An in-column energy (omega) filter and a 25 eV slit were used. The images were recorded via SerialEM software under low-dose conditions onto a bottom-mounted TemCam-F416 camera (TVIPS).

Each sample was prepared using an automatic plunge-freezer system (Leica EM GP) with the environmental chamber operated at 20 °C and 90% relative humidity. A droplet (4 μL) taken from a sample that had been incubated at 37 °C was deposited on a lacey Formvar carbon-coated grid (Ted Pella) and was blotted with filter paper to remove excess fluid. The grid was then plunged into liquid ethane (around −184 °C) to ensure the rapid vitrification of the sample in its native state. Prior to the cryo-TEM measurements, the specimens were stored in liquid nitrogen (−196 °C) before imaging under the microscope using a cryotransfer tomography holder (Fischione Model 2550).

### Kinetics of Fibril Formation

The kinetics of fibril formation were studied at 37 °C using the fluorescent dye thioflavin T (ThT) as a reporter of fibril formation. Protonated aSyn in 10 mM MES pH 5.5 prepared in H_2_O was incubated in a PEG-ylated 96-well plate (Corning 3881) under quiescent conditions in a plate reader (Fluostar Omega). The ThT fluorescence was measured through the bottom of the plate every 60 s with a 448 nm excitation filter and 480 nm emission filter. The initial monomer concentration was 0.40 mM, the seed concentration 4 μM, the ThT concentration 20 μM, and the lipid concentration 2.1 mM.

## Results and Discussion

In this study, we have investigated how lipid bilayer discs respond to the adsorption of aSyn monomers and/or aSyn fibril formation occurring in their presence. Lipid bilayer discs are heterogeneous with respect to their curvature. They are composed of two distinct regions, the flat part and the rim where the lipid monolayer needs to curve to avoid a significant hydrocarbon–water contact. Because of the structural constraints at these two very different sites, lipid bilayer discs are often required to contain a mixture of two lipid components to be stable. One (typically the major) lipid component has a preference for the flat regions, and the other lipid component has a preference for curved regions.^33^ The lipids preferring a flat bilayer are generally zwitterionic phospholipids with two long acyl chains, while the lipids preferring the curved rims tend to be, for example, short-chain lipids,^34−36^ ganglioside GM1 lipids,^37^ or PEGylated lipids.^38,39^ In the present study, we have used a lipid mixture composed of DMPC and DMPS, at the molar ratio DMPC/DMPS = 7/3. Here, the charged lipid, DMPS, is believed to have a preference for the curved rim because of the contribution from electrostatic interactions to the monolayer spontaneous curvature.^33^ The intrinsic p*K*_a_ of the carboxylate group of PS is approximately 3.6.^40^ However, because of the negative surface potential, the apparent p*K*_a_ (defined as the bulk pH when half of the carboxyl groups are dissociated) is higher. Due to the inhomogeneous distribution of DMPS, it is difficult to estimate the exact degree of protonation, but it is possible that a significant fraction of the carboxyl groups are protonated at the present solvent conditions.

**Figure 1:**
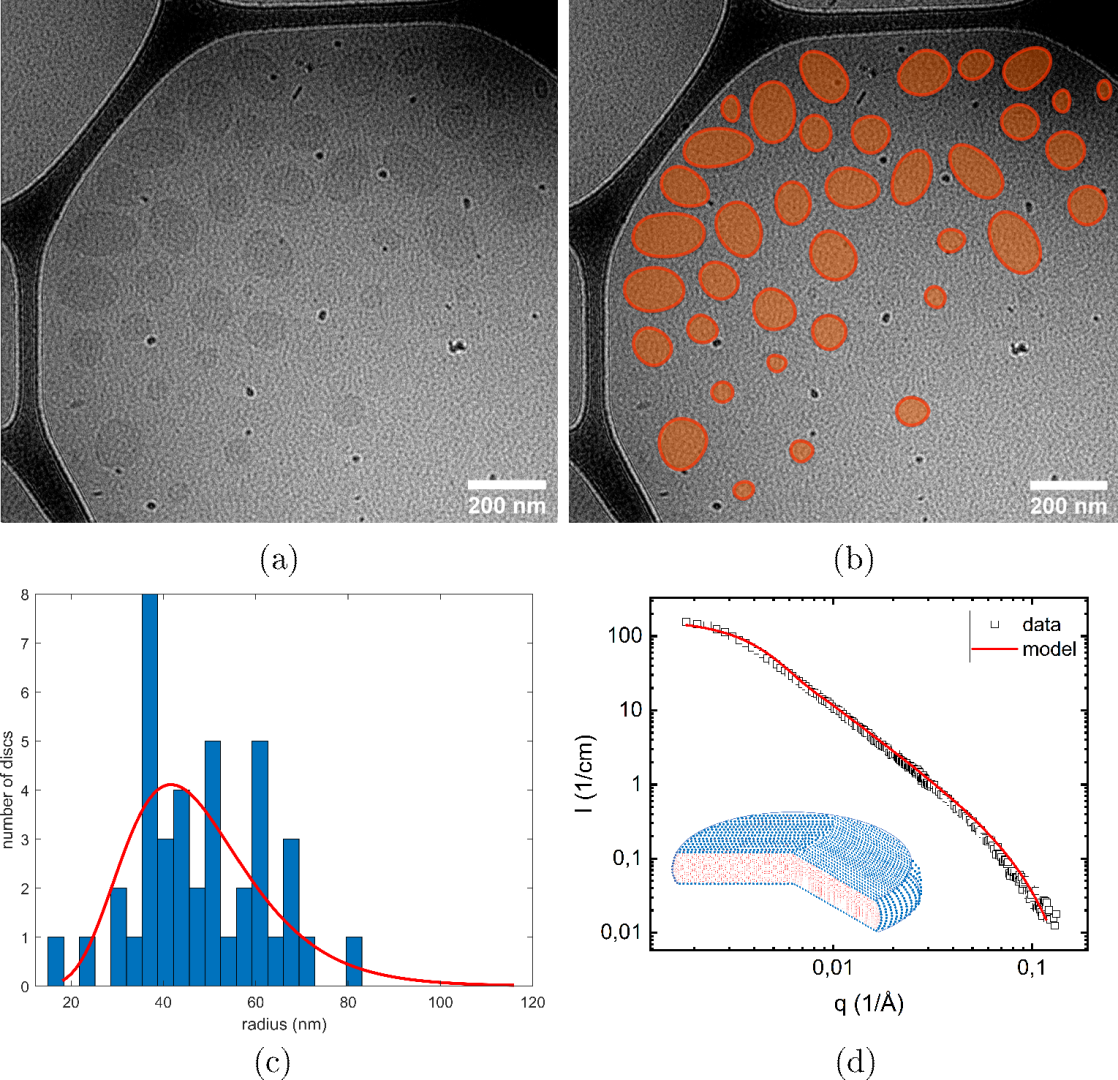
(a) Cryo-TEM image of a lipid bilayer disc suspension at a lipid concentration of 7.5 mM. (b) The same image as shown in panel (a) with discs highlighted with orange color for better visualization. (c) Size distribution of the lipid bilayer disc radius obtained from cryo-TEM images compared with a log-normal distribution (red line). The diameter of the tilted discs was set to be the longest axis. (d) Scattering profile of a 2.1 mM disc dispersion (black squares) and the disc model that provides the best agreement with the data (red line). The inset shows a schematic illustration of a lipid bilayer disc, where the red color indicates the lipid chain region, and the blue filled circles represent the lipid headgroups.

In Figure 1a, we present a cryo-TEM image of a pure lipid bilayer disc sample having a lipid concentration *c*_L_ = 7.5 mM in 10 mM MES buffer at pH 5.5. The presence of disclike structures, rather than vesicles, is confirmed by the absence of sharp contrast at rims, as expected for hollow vesicles.^41−43^ In Figure 1b, we show the same image but with the lipid bilayer discs highlighted with an orange color. The lipid bilayer discs are close to circular in shape, as is expected due to a finite line tension of the rim, which favors a circular shape. However, several discs deviate from a circular shape in the 2D image projection, which we interpret as different disc orientations. When the disc normal is not perpendicular to the plane of view, circular discs appear elliptic, and as a line when the normal is in the plane (Figure S2). Assuming circular discs, one may still evaluate the disc diameter from the longest axis of the elliptical shape. We have made a coarse analysis of the disc size distribution observed by cryo-TEM (Figure 1a). The resulting histogram, compared to a log-normal distribution curve, is shown in Figure 1c, yielding an average size of ⟨*R*⟩ = 50 nm.

The relative stability of vesicles and discs has been analyzed previously.^44−47^ In short, discs are stable for smaller areas while vesicles are the stable structure for larger areas. The transition occurs at a disc radius approximately given by *R* ≈ 8κ/γ, where κ is the bending rigidity, and γ is the line tension.^47^ Thus, discs are favored over vesicles in the case of a high bending rigidity and a low line tension.

In Figure 1d, we present a SANS pattern from the lipid bilayer discs at *c*_L_ = 2.1 mM. Neglecting interactions between discs, which is a reasonable approximation due to the low lipid concentration and the 10 mM buffer acting as a screening electrolyte, the scattering profile, *I*(*q*), can be written as
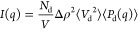
1Here,  is the number density of discs, *V*_d_ the disc volume, Δρ the scattering length density difference between discs and the solvent, and *P*_d_(*q*) the particle form factor. The brackets ⟨...⟩ denote ensemble averages over polydisperse discs. For a circular disc of radius *R* and thickness *t*, the orientationally averaged form factor is given by^48^
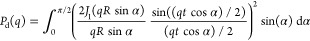
2The model fitting was done using SasView (https://www.sasview.org/) software, with a fixed disc (bilayer) thickness *t* = 3.7 nm and Δρ = 6 × 10^–6^ Å ^–2^. The best fit is shown in Figure 1d as a solid red line and corresponds to *R* = 51 nm ± 15 nm where 15 nm is the standard deviation assuming a log-normal distribution in *R*. Regarding the size distribution of the lipid bilayer discs, a good agreement between cryo-TEM and SANS results was found.

As mentioned above, we expect the charged lipid, DMPS, to have a preference for the curved rim of a disc. In order to calculate the relative area of the curved rim, we modeled the lipid bilayer discs as flat circular discs of radius *R* and thickness *t*, with the rim further coated with a curved lipid film, as illustrated in the inset in Figure 1d. The interfacial area of the flat part is *A*_f_ = 2*πR*^2^, while the area of the curved rim can be approximated by *A*_c_ = π^2^*Rt*. The area fraction of the curved rim is then *A*_c_/*A* = *πt*/(*πt* + 2*R*), where *A* = *A*_c_ + *A*_f_. The present lipid bilayer discs have an average radius *R* ≈ 50 nm and *t* ≈ 4 nm, giving *A*_c_/*A* ≈ 0.1. This value is smaller than the DMPS molar fraction [DMPS]/([DMPS] + [DMPC]) = 0.3, implying that there is an excess of charged lipids with respect to the curved rim area that can act as a reservoir giving a sufficiently low line tension to stabilize the lipid bilayer discs. Note that while we expect DMPS to accumulate at the charged rim, we do not expect the rim to be completely DMPC free. The equilibrium concentration of DMPS in the rim is dependent on the balance between the electrostatic free energy, the entropy of mixing, and the local DMPC–DMPS lateral interactions. In any case, having an excess of DMPS compared to what is needed to stabilize the rim implies that the corresponding line tension may be sufficiently small to also allow for deformation of discs.

We note also that the SANS pattern and cryo-TEM images are consistent with flat discs, and this is expected because of the high bending rigidity of phospholipid bilayers, κ = 10*k*_B_*T*–20*k*_B_*T*.^49^ De Gennes derived that the persistence length of a semiflexible surface is given by^50^
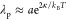
3where *a* is the molecular size, of the order of 1 nm. With κ = 10*k*_B_*T*–20*k*_B_*T*, we obtain λ_p_ ≫ *R*.

### Lipid Bilayer Discs and α-Synuclein

Having characterized the lipid bilayer discs in the absence of protein, we now proceed to characterize the discs in the presence of aSyn. We used deuterated aSyn and D_2_O buffer, in which case the protein is essentially contrast-matched to the buffer, and the SANS pattern reports only on the lipid bilayer discs. The experiments were performed at the lipid-to-protein ratio L/P = 5 in 10 mM MES D_2_O buffer (pH 5.5) and at 37 °C. At this mildly acidic pH, which can be found in certain cellular compartments such as endosomes,^51,52^ new fibrils rapidly form by a secondary nucleation mechanism in the presence of already formed fibrils.^53^ For this reason, we added aSyn fibril seeds at a concentration of 4 μM (1 mol % of the total protein concentration) in order to ensure rapid aSyn aggregation.

**Figure 2:**
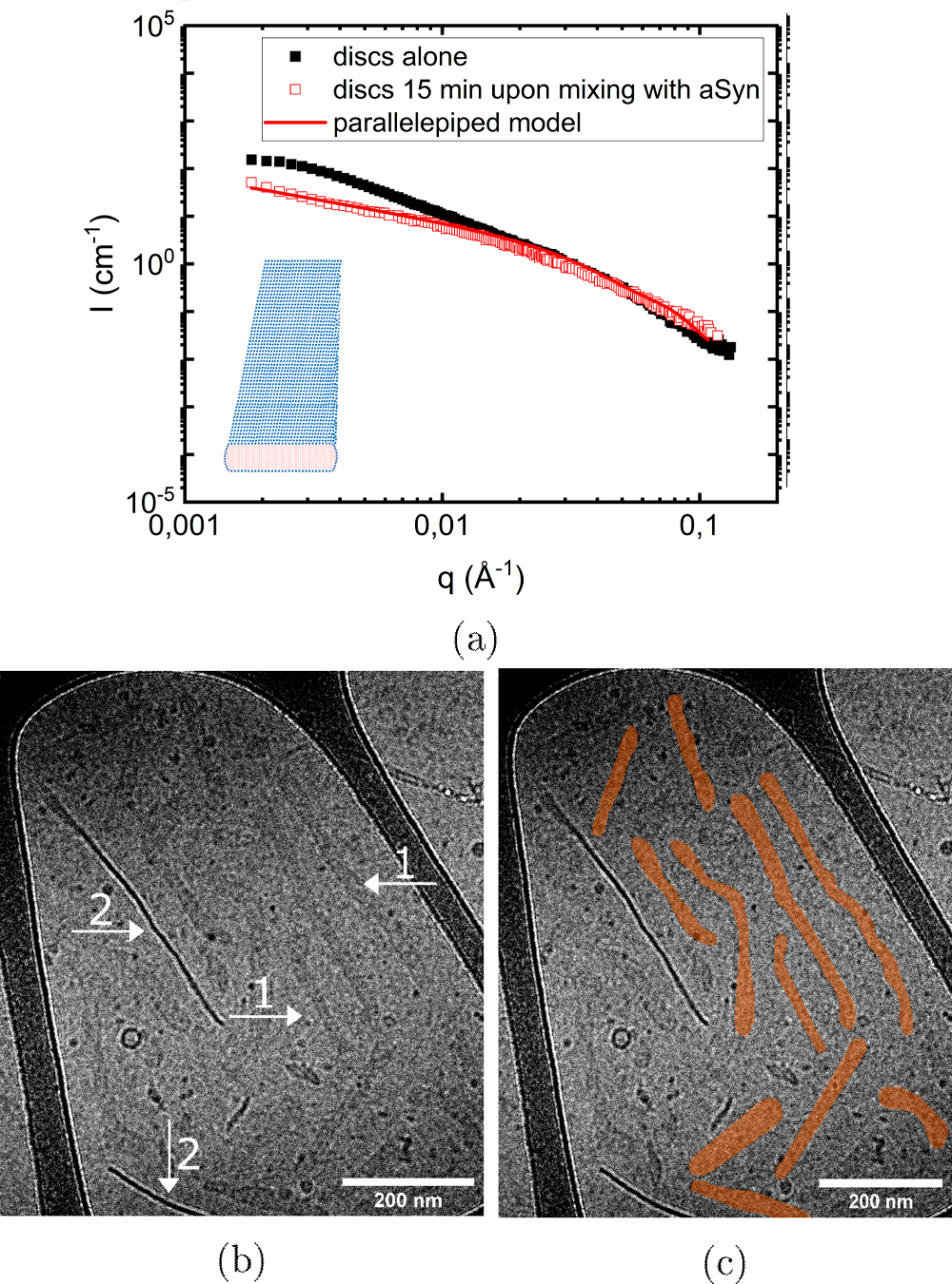
(a) Scattering profiles of a pure disc dispersion (filled black squares), of discs during the first 15 min after addition of aSyn (open red squares), and the parallelepiped model (solid red line). The best model was obtained with a length of shorter edge *a* = 13 nm and a length of longer edge *b* = 550 nm. The inset shows an illustration of the parallelepiped, where the red color illustrates the lipid chain region, and the blue represents the lipid head groups. (b) Cryo-TEM image taken 13 min after mixing lipid bilayer discs and aSyn. Arrows labeled with number 1 indicate elongated structures, and arrows labeled with number 2 indicate elongated structures whose normal is not perpendicular to the plane of view. (c) The same image as shown in panel b with deformed lipid bilayer discs highlighted in orange for better visualization. Scale bars in panels b and c correspond to 200 nm. The protein and lipid concentrations in all of the samples were 0.40 and 2.1 mM, respectively.

In Figure 2a, we show the recorded SANS pattern (ca. 15 min after protein addition) together with a SANS pattern recorded in the absence of aSyn. As can be seen, there is a significant difference in the patterns at lower *q*-values between the two samples, which is a consequence of a change in the particle form factor. In this low-*q* regime, *q* < 0.02 Å ^–1^, the intensity has significantly decreased in the sample with aSyn. For the pure lipid bilayer discs, an *I*(*q*) ∼ *q*^–2^ dependence of scattered intensity, a signature of scattering from 2D objects, was observed. However, when the lipid bilayer discs are mixed with aSyn, that dependence changes to *I*(*q*) ∼ *q*^–1^, indicating that the originally circular lipid bilayer discs have become highly elongated.

We have modeled the deformed discs as simple parallelepipeds, and the model that best agrees with data is shown as a solid red line in Figure 2a. The orientationally averaged form factor of a parallelepiped, *P*_p_(*q*), is given by^48^
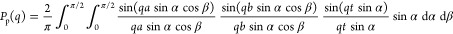
4

The integration is performed in order to account for all possible orientations.^54^ Here, α is an angle between the parallelepiped’s longest axis and *z*-axis of the coordinate system whose origin is located at the parallelepiped’s center, and β is an angle between the scattering vector  and the *y*-axis of the same coordinate system.

The model fitting was done using SasView (https://www.sasview.org/) software, with a fixed (bilayer) thickness *t* = 3.7 nm and Δρ = 6 × 10^–6^ Å ^–2^. We denote the other two parallelepiped sides by *a* and *b*. A good agreement between the model and the data is obtained with *a* = 13 nm and *b* = 550 nm. A parallelepiped lipid bilayer disc is illustrated in the inset of Figure 2a.

The elongated disc shape was confirmed using cryo-TEM. A representative image is shown in Figure 2b. The sample was vitrified ca. 13 min after mixing the lipid and protein. Highly elongated lipid bilayer discs, with the length *b* being several 100 nm, can be seen, although again with low contrast. To guide the eye, we have highlighted the objects by giving them an orange color in Figure 2c.

It is well established that aSyn molecules adsorb onto negatively charged lipid membranes, and we attribute the observed disc shape deformation to the adsorption of aSyn. From the observed dimensions of the deformed lipid bilayer discs in the cryo-TEM image, it appears that the deformation of lipid bilayer discs occurs at constant overall disc size (lipid aggregation number), meaning that the total interfacial area, *A* = *A*_f_ + *A*_c_, remains essentially unchanged. The observed deformation then implies that the area of the curved rim *A*_c_ = π(*a* + *b*)*t* has increased at the expense of the flat part *A*_f_ = 2*ab*. With the *a* and *b* values obtained from the SANS data, we find *A*_c_/*A* ≈ 0.3, which is an increase by a factor of 3 compared to the original circular disc. This suggests that the aSyn molecules have a preference for adsorbing at the curved rim, compared to the flat part, presumably because of a higher charge density at the rim. We note that while there is a preference of aSyn to adsorb at the highly charged rim, we still expect also some adsorption to the flat bilayer part. In a simple picture of the thermodynamics, the shape deformation and elongation of the rim are expected to involve a free energy penalty in the form of an increase in the line energy, Δ*G*_l_ = γΔ*l* > 0, where Δ*l* is the increase of the length, *l*, of the disc rim. However, this is then compensated for by a spontaneous adsorption of aSyn molecules, with Δ*G*_a_ < 0. The equilibrium deformation is then characterized by Δ*G*_l_ + Δ*G*_a_ = 0.

Here, the lipid-to-protein ratio, L/P = 5, is relatively low, and we expect to have a significant fraction of free, nonadsorbed aSyn molecules.^17^ The excess of the free protein allows for the fibril formation to take place, as discussed in Galvagnion et al.^23^

**Figure 3:**
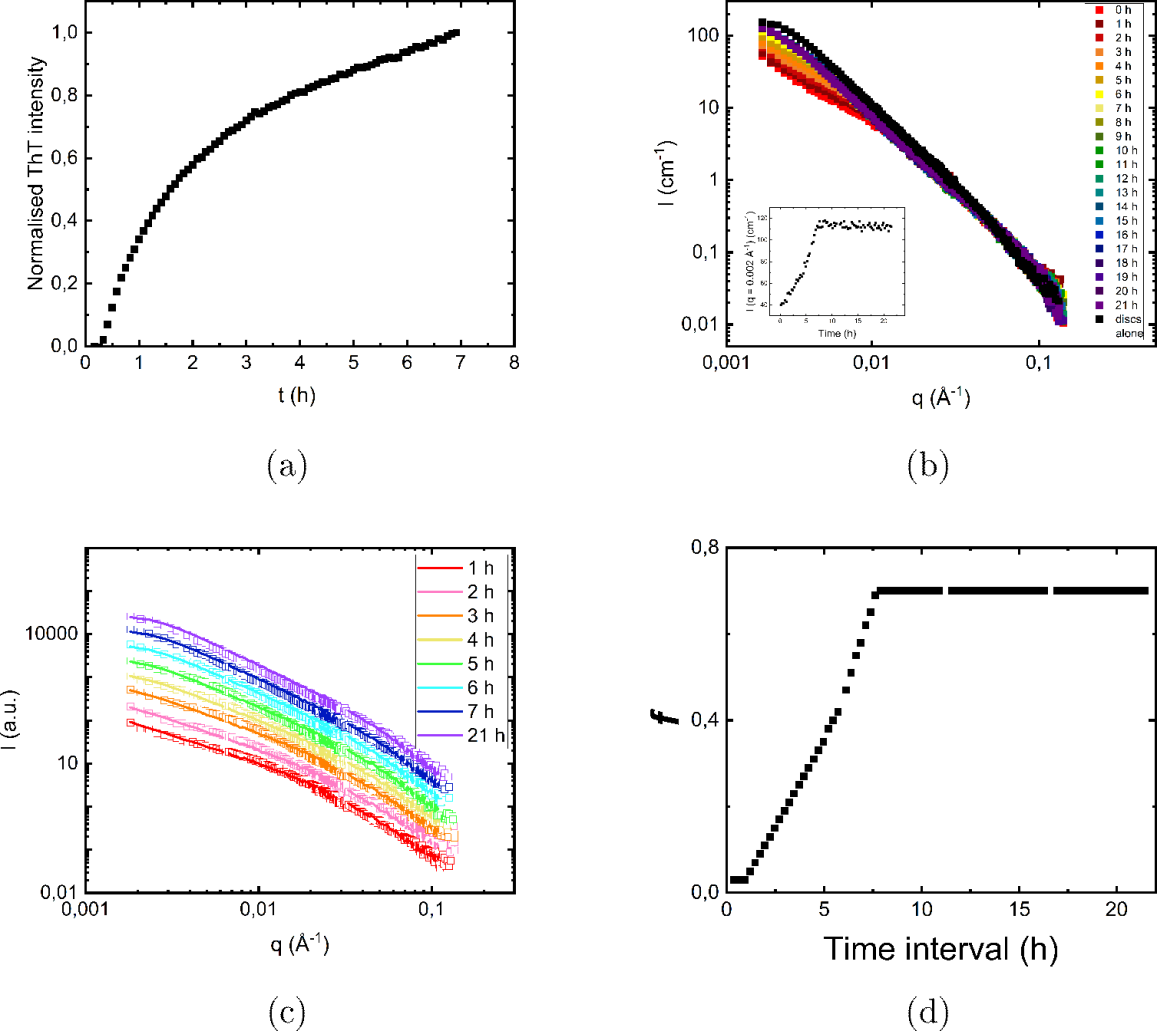
(a) ThT fluorescence intensity versus time. (b) Time-resolved SANS profiles, *I*(*q*) vs *q*, plotted for a total time period of ca. 22 h. with a time resolution of 1 h. The inset shows a plot of the intensity obtained at *q* = 0.002 Å ^–1^ as an additional illustration of the time evolution of the scattered intensity. (c) Scattering profiles obtained at different time points (open squares) were modeled as a superposition (solid lines) of the scattering profile obtained during the first 15 min after mixing and the pure disc dispersion. The data are shifted for easier comparison. (d) Fraction of undeformed circular lipid bilayer discs versus time. The protein and lipid concentrations in all of the samples were 0.40 mM and 2.1 mM, respectively.

In order to follow how the lipid bilayer discs changed over the course of the aSyn aggregation process, we performed a 22 h long time-resolved contrast-matching SANS experiment. Prior to the SANS experiment, we conducted a ThT assay at the same conditions as used in the SANS experiment (pH 5.5 and 37 °C, *c*_L_ = 2.1 mM and *c*_P_ = 0.4 mM), in order to gain more insight in the kinetics of aSyn fibril formation. Figure 3a shows the time dependence of the recorded fluorescence intensity, which acts as an indicator of fibril formation. As can be seen, there is a short lag time of ca. 15 min after which the fluorescence intensity rapidly increases with time.

In Figure 3b, we present time-resolved SANS patterns, recorded over a time period of 22 h with a time resolution of 1 h, and compare them to the scattering profile of a pure disc dispersion. The large difference between scattering patterns of protein-free and aSyn-containing samples, observed at early time points, gradually decreases with time as the scattering pattern of protein–lipid samples approaches the pattern of the original circular discs, reaching a steady state after ca. 8 h. The recovery to the initial, circular shape is most likely associated with desorption of aSyn molecules from the surface of the lipid bilayer discs. As an inset of Figure 3b, we are showing the scattering intensity recorded at *q* = 0.002 Å ^–1^. An apparent lag time of ca. 1 h is observed, after which the intensity increases with time and reaches a steady state value after ca. 8 h.

Interestingly, the scattering patterns at intermediate time points are well described by a linear combination of the scattering profile obtained from the pure disc dispersion, *I*_d_(*q*), and the scattering profile obtained in the first 15 min after mixing discs and protein, *I*_15min_. The linear combination is calculated as *I*(*q*) = *fI*_d_ + (1 – *f*)*I*_15min_, where *f* is then the fraction of circular lipid bilayer discs, and 1 – *f* is the fraction of elongated discs. The fact that the scattering patterns obtained at later time points are successfully fitted with the linear combination of circular and elongated lipid bilayer discs implies that there is coexistence of the recovered and elongated discs, which suggests that aSyn desorption is a cooperative process.

In Figure 3c, we show some selected scattering patterns together with the linear combination fits, and in Figure 3d, we have plotted the obtained *f*-values as a function of time. As can be seen in Figure 3d, *f* reaches a steady state value of 0.7 after ca. 8 h. Thus, 70% of the lipid bilayer discs had recovered their original circular shape, while the other 30% remained as highly elongated as they were at the early time points.

**Figure 4:**
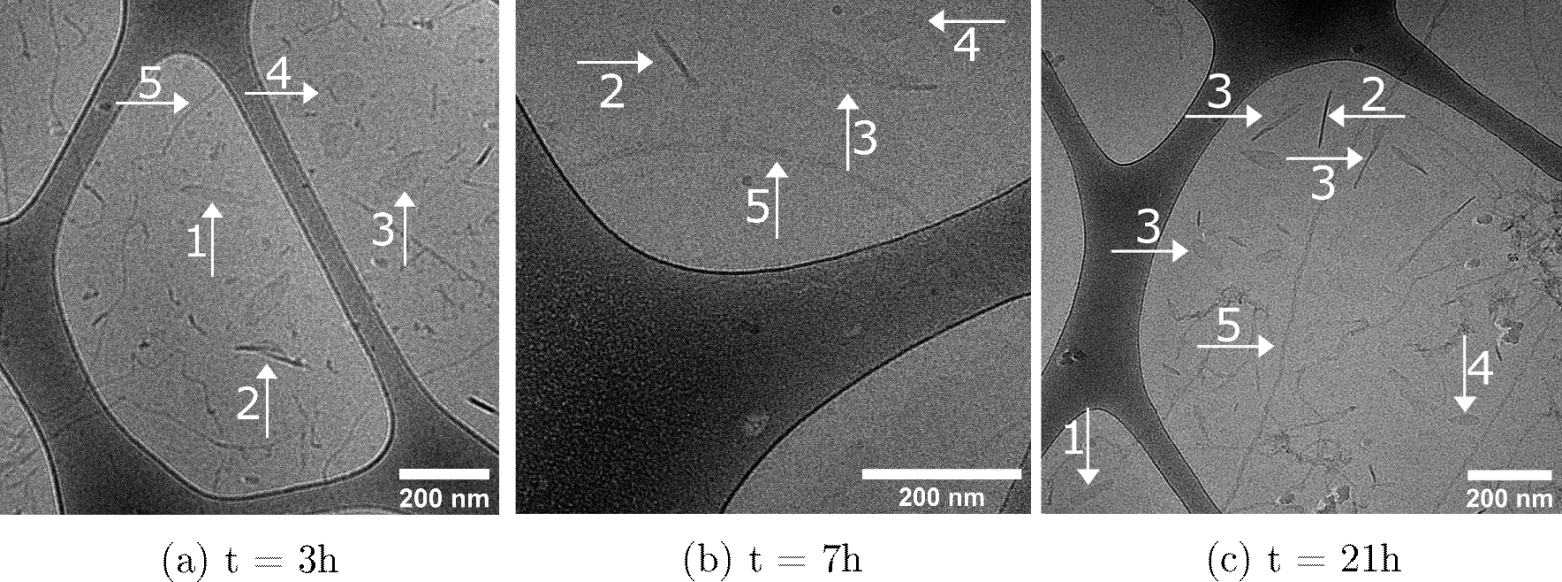
Cryo-TEM images taken (a) 3 h after mixing, (b) 7 h after mixing, and (c) 21 h after mixing. Arrows labeled with the number 1 indicate elongated structures, arrows labelled with the number 2 elongated structures whose normal is not perpendicular to the plane of view, arrows labeled with the number 3 partially recovered structures, arrows labeled with the number 4 structures recovered to the circular shape, and arrows labeled with the number 5 aSyn fibrils. More cryo-TEM images are shown in the SI (Figure S3).

In Figure 4, we show cryo-TEM data from samples containing protein and lipid bilayer discs at different time points, 3, 7, and 21 h, after mixing. In images taken at all of these time points, we see structures consistent with the recovery from elongated objects, partially recovered structures, as well as the presence of elongated structures, in agreement with the cooperative desorption mechanism as inferred from the SANS data. We note that the partially recovered structures present in these figures are not stable but rather that the sample snapshot was taken while not all of the monomers were desorbed from the partially recovered disc.

Due to fibril formation, there is a desorption of aSyn molecules from the surface of lipid bilayer discs. However, when investigated in separate containers on separate instruments, there is a slight discrepancy in the recorded time-dependencies of desorption and fibril formation. This is not unexpected as the aggregation kinetic depends on various factors including pH,^53^ temperature,^55^ the presence of an air–water interface,^56^ the nature of the surface of the sample container,^57^ in this case PEGylated polystyrene plate (ThT assay) versus quartz cells (SANS experiments), and the mode of container handling during the measurement.

**Figure 5:**
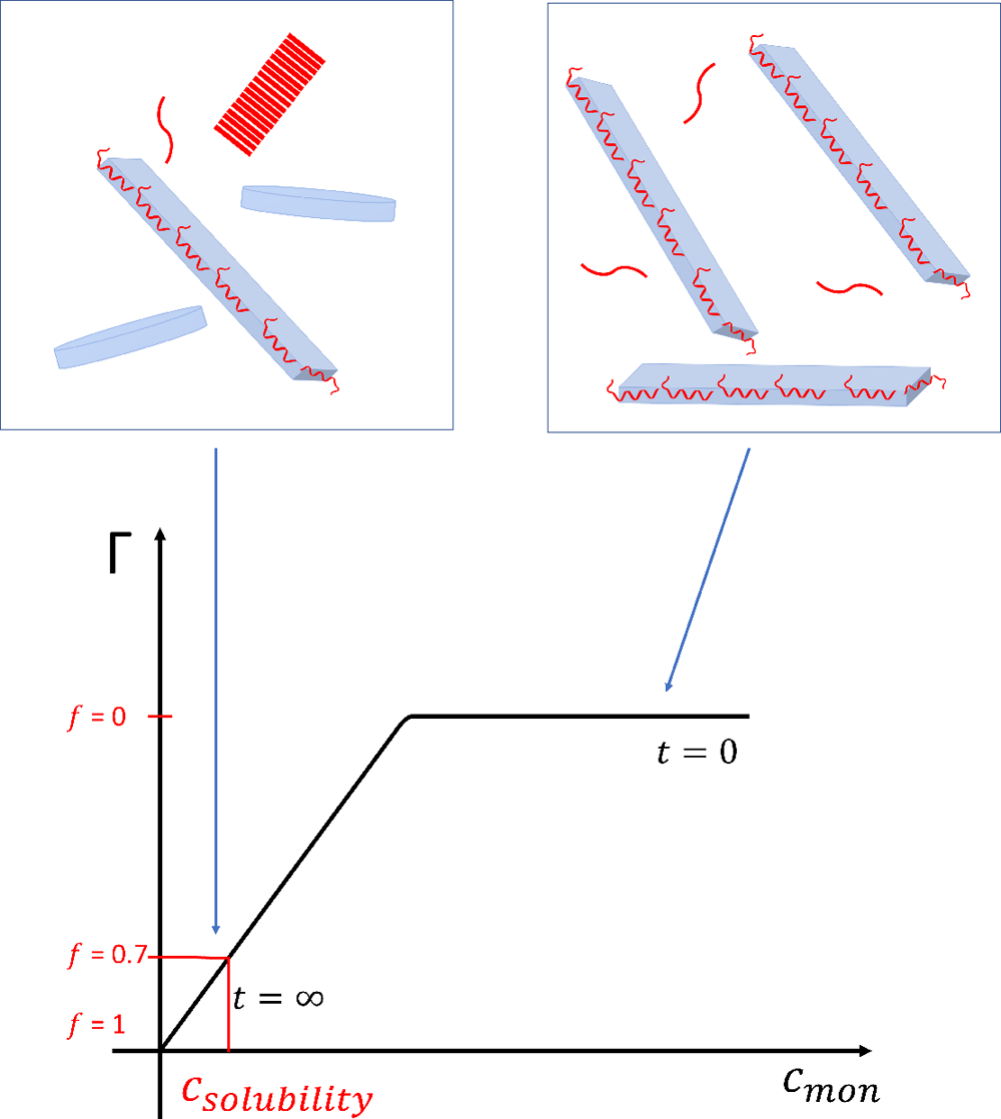
Illustration of adsorbed amount, Γ, versus monomer concentration. The recovery parameter reaches its equilibrium value *f*_eq_ = 0.7 at the monomer concentration which corresponds to the solubility value.

The proposed process of monomer desorption can be discussed in terms of the adsorption isotherm illustrated in Figure 5. At *t* = 0, all discs (in blue) are covered with aSyn monomers (in red), and they are elongated. The fibril formation, which occurs at later time points, is followed by desorption of monomers from the lipid bilayer discs and their incorporation into aSyn fibrils (in red). As there is nonzero monomer concentration in equilibrium with fibrils, there are still monomers adsorbed onto the disc surface, resulting in *f* < 1.

The fact that the SANS patterns at different time points are well described by a linear combination of fully deformed lipid bilayer discs and unperturbed circular lipid bilayer discs is striking. This implies that, at a given time point, two populations of discs coexist: one population of discs that are deformed and presumably saturated with the maximum possible adsorbed amount of aSyn, and a second population of undeformed circular lipid bilayer discs, presumably having no aSyn molecules adsorbed. With time, only the relative fraction of each population is changing. A similar scenario was described in a recent paper,^17^ where Makasewicz et al. systematically studied the adsorption of aSyn onto mixed zwitterionic–anionic lipid vesicles and found it to bind in an all-or-none fashion. Completely covered vesicles were found to coexist with vesicles that did not have any bound aSyn. The main conclusion of that paper was that the adsorption process is cooperative. Interestingly, in the present experiment where aSyn gradually desorbs, essentially the same all-or-none binding to the lipid bilayer discs is observed. The coexistence of discs with saturated adsorption and discs with no adsorption implies that discs with intermediate adsorption are unstable with respect to the limiting states. The molecular mechanism behind this very strong cooperativity remains to be found.

Finally, we note that the deformation of lipid bilayer discs and recovery to their original shape appear to occur at a constant number of discs, with a constant aggregation number distribution. We base this on the fact that the forward scattering, *I*(0), at steady state appears to be very similar to *I*(0) obtained from undeformed circular lipid bilayer discs, in the absence of protein. This implies that there is no disc clustering or disc fusion.

## Conclusions

In this study, we investigated the interaction of aSyn and lipid bilayer discs composed of DMPC/DMPS lipids, with 30% of charged lipids. We believe that the charged, DMPS, lipids accumulated at the curved rims, and that the DMPC lipids preferentially populate the flat part of the discs. However, there is still a significant fraction of charged lipids in the flat part.

**Figure 6:**
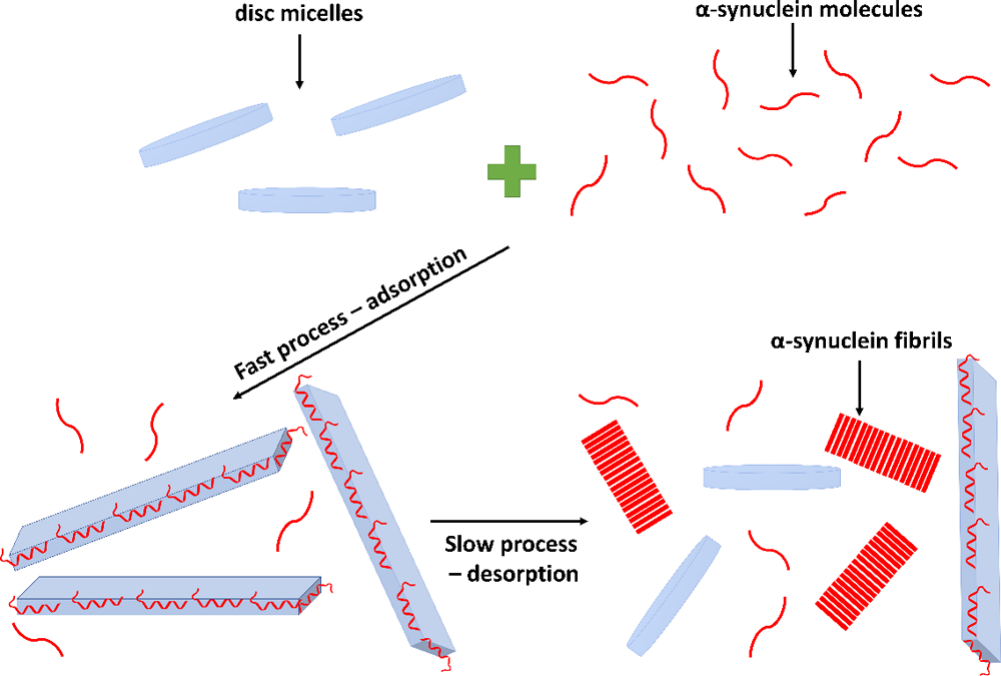
An illustration of the overall events suggested by the experimental data. Top: circular lipid bilayer discs (blue) are incubated with aSyn monomers (red). Bottom left: adsorption of aSyn monomers onto lipid bilayer discs is a fast process that results in formation of elongated structures that can be represented by parallelepipeds. Bottom right: aSyn desorption is a cooperative and slow process that results in a recovery of discs to their original shape.

The main findings of this paper are summarized in Figure 6. The adsorption of aSyn monomers onto the lipid bilayer disc surface results in a striking change of the morphology of the discs. The initially circular discs assume highly elongated shapes. In this shape transition, the length of the curved rim increases 3–4 times. The fact that the area of the curved part increases while the area of the flat part decreases implies that aSyn has a preference for the curved part. When fibril formation is initiated, the monomers appear to desorb from the disc surface to instead be incorporated into the fibrils leading to the disc recovering to its initial circular shape. By analyzing the SANS and cryo-TEM data obtained at various time points, we find that the desorption is cooperative, as was previously shown for aSyn adsorption to lipid bilayers.^17^

## Supporting Information Available

Scattering profile of aSyn monomers at pH 11, additional cryo-TEM image of a lipid bilayer disc suspension with the presence of discs whose normal is not perpendicular to the plane of view, and additional cryo-TEM images taken 3 and 7 h after mixing protein and lipid bilayer discs (PDF)
