# Supplementary material for: α-Synuclein Interaction with Lipid Bilayer Discs
Source: Langmuir. 2022 Aug 11;38(33):10216–24. doi: 10.1021/acs.langmuir.2c01368 (PMC9404543; doi:10.1021/acs.langmuir.2c01368)
Supplement: Supplementary file 1 — la2c01368_si_001.pdf [file la2c01368_si_001.pdf]

Supplementary information: α-Synuclein interaction with lipid bilayer discs

Marija Dubackic,[*,†] Yun Liu,[‡,¶] Elizabeth G. Kelley,[‡] Crispin Hetherington,[§] Michael Haertlein,[||] Juliette M. Devos,[||] Sara Linse,[⊥] Emma Sparr,[†] and Ulf Olsson[†]

†*Physical Chemistry, Chemistry Centre, Lund University, SE-22100 Lund, Sweden*

‡*Center for Neutron Research, National Institute of Standards and Technology, Gaithersburg, MD 20878, United States*

¶*Chemical and Biomolecular Engineering Department, University of Delaware, Newark, DE 19716, United States*

§*National Center for High Resolution Electron Microscopy, Centre for Analysis and Synthesis, Chemistry Centre, Lund University, SE-22100, Lund Sweden*

||*Life Sciences Group, Institut Laue-Langevin, 38000 Grenoble, France*

⊥*Biochemistry and Structural Biology, Chemistry Centre, Lund University, SE-22100 Lund, Sweden*

E-mail: marija.dubackic@fkem1.lu.se

Phone: +46-46-222 81 85

In Figure S1 we are showing aSyn dissolved at pH 11.4. The data was fitted with Debye function given by[1]

$$I(q) = I_0 2(e^{-(qR_g)^2} + (qR_g)^2 - 1)/(qR_g)^4 + BG, \tag{1}$$

where $R_g = 4$ nm is monomer radius of gyration, $I(0)$ is the intensity at $q = 0$ and BG is the background term. The fact that the data was successfully fitted with Debye function which describes scattering of a random coil, shows that aSyn was in its monomeric form prior to addition of lipid bilayer discs.

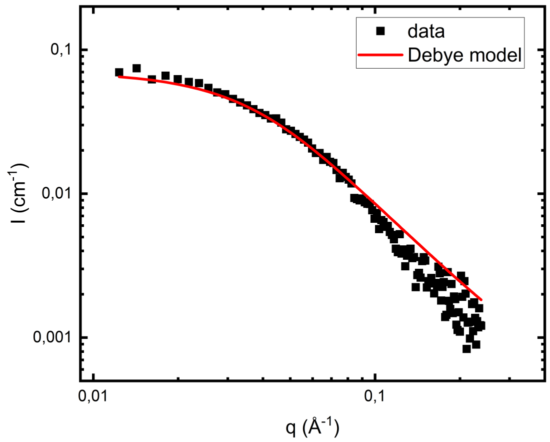

Figure S1: Scattering profile of α-synuclein at pH 11.4 (black dots) and the random coil model (red line). The protein concentration is 280 μM (where M = mol/L denotes molar concentration) and the model that best represents data was obtained for radius of gyration equal to 4 nm.

In Figures S2 and S3 we are showing additional cryo - TEM images. In Figure S2, we are showing an image of the pure disc suspension where the presence of discs whose normal is not perpendicular to the plane of view is observed. In Figure S3, we are showing elongated structures present 3 and 7 hours after addition of aSyn to lipid bilayer disc dispersion.

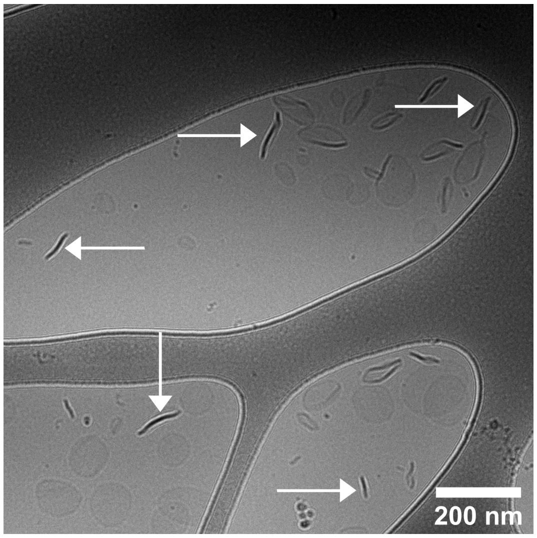

Figure S2: Cryo – TEM image of lipid bilayer disc suspension at lipid concentration of 7.5 mM. White arrows indicate discs that have normal which is not perpendicular to the plane of view.

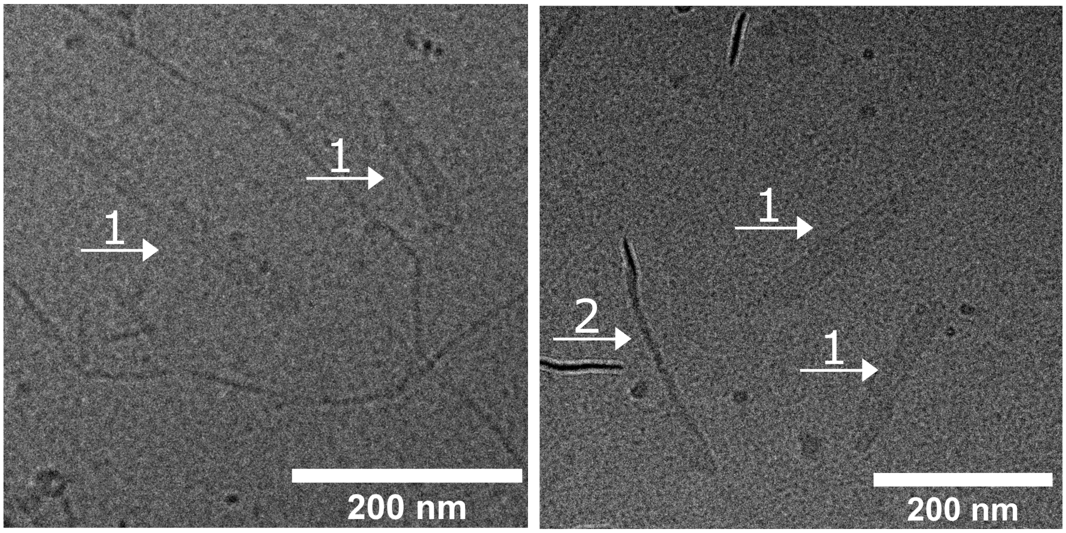

(a)

(b)

Figure S3: Cryo - TEM images of extended structures present 3 h (a) and 7 h (b) after mixing lipid bilayer discs and aSyn. Arrows labeled with number 1 indicate elongated structures and arrows labeled with number 2 indicate elongated structures whose normal is not perpendicular to the plane of view.

References

(1) Pedersen, J. S. Analysis of small-angle scattering data from colloids and polymer solutions: modeling and least-squares fitting. *Adv. Colloid Interface Sci.* **1997**, *70*, 171–210.
